# Metastatic Disease in Phaeochromocytomas and Paragangliomas in Various Genotypes—A Systematic Review and Meta-analysis

**DOI:** 10.1210/clinem/dgaf584

**Published:** 2025-11-03

**Authors:** Louise Kirkegaard Svendsen, Åse Krogh Rasmussen, Marianne Christina Klose, Jesper Krogh, Ulla Feldt-Rasmussen

**Affiliations:** Department of Nephrology and Endocrinology, Copenhagen University Hospital: Rigshospitalet, Copenhagen 2100, Denmark; Department of Nephrology and Endocrinology, Copenhagen University Hospital: Rigshospitalet, Copenhagen 2100, Denmark; Department of Nephrology and Endocrinology, Copenhagen University Hospital: Rigshospitalet, Copenhagen 2100, Denmark; Clinic for Pituitary Disorders, Zealand University Hospital, Køge 4600, Denmark; Department of Nephrology and Endocrinology, Copenhagen University Hospital: Rigshospitalet, Copenhagen 2100, Denmark

**Keywords:** paraganglioma, pheochromocytoma, metastasis, genotype, systematic review, meta-analysis

## Abstract

**Background:**

Paragangliomas and phaeochromocytomas (PPGLs) are rare neuroendocrine tumours with 10% to 20% of patients developing metastatic disease. The tumors exhibit a high degree of heritability, and pathogenic genetic variants have been associated with metastases.

**Objective:**

We aimed to investigate the association between the genotype of PPGL patients and their risk of metastatic disease, adjusting for time.

**Methods:**

A systematic search was conducted in PubMed and Embase. The primary outcome was the rate of metastatic disease per 100 followed patient-years analyzed through meta-analyses.

**Results:**

A significantly increased rate of metastatic disease per 100 followed patient-years was observed in all pathogenic germline variants included in the analyses, compared to the group with no identified variant. The group with no variant had a rate of 1.55 per 100 patient-years. *SDHA, SDHC, SDHD, VHL, RET, NF1*, and *MAX* had rates of 13.73, 6.27, 2.03, 2.34, 1.91, 4.11, and 9.66, respectively. The rate of *SDHB* is not presented as statistical heterogeneity exceeded 75%. The pathogenic somatic variant *EPAS1* showed a rate of 3.82. Cluster-divided meta-analyses resulted in rates of 4.41 and 3.0 for cluster 1 and cluster 2, respectively. Meta-regression analysis revealed a 2.3-fold higher rate for the *SDHB* variant compared to the other cluster 1 variants.

**Conclusion:**

We present associations between genotype and metastatic disease in PPGL patients. Our results indicate that patients harboring a pathogenic genetic variant have a higher rate of metastases compared to patients with no identified variant. High heterogeneity in several analyses suggests that further large cohort studies are needed.

According to the 2017 World Health Organization classification, paragangliomas (PGL) are extra-adrenal tumours originating from specialized neuroendocrine cells derived from the neural crest in the autonomic nervous system ganglia and associated nerves. PGLs can be located in the sympathetic or parasympathetic system. Phaeochromocytomas are intra-adrenal sympathetic paragangliomas. Phaeochromocytomas and paragangliomas are collectively abbreviated as PPGL (1).

PPGLs can hold a wide range of pathogenic germline and/or somatic genetic variants, and they exhibit the highest known heritability rate among all human tumors. Studies have found a pathogenic germline variant in up to 40% of patients and a pathogenic somatic variant in 25% to 30% (2). These variants can be grouped into 3 main molecular clusters based on which underlying signaling pathway the alteration affects. Cluster 1, known as the pseudohypoxic cluster, encompasses variants affecting either the Krebs cycle (cluster 1A) or hypoxia signaling (cluster 1B). These variants result in DNA hypermethylation and inactivation of tumor suppressor genes, causing hypoxia-inducible factor-α stabilization and subsequently angiogenesis, tumor extravasation, migration, invasion, and metastases. Cluster 2, referred to as the tyrosine kinase-linked signaling pathway, is characterized by variants that activate pathways such as PI3 K/AKT, RAS/RAF/ERK, and mTORC1 but also, like cluster 1, increased synthesis of hypoxia-inducible factor-α. Clusters 1 and 2 comprise both germline and somatic pathogenic variants. Cluster 3 variants are solely somatic but are less studied than the other 2. Cluster 3 is also called the Wnt-signaling-related cluster and leads to overactivation of Wnt- and β-catenin signaling, resulting in tumor angiogenesis, proliferation, invasion, and metastases (3).

Previous understanding of PPGLs was based on the concept of tumors as either benign or malignant. This has changed as the World Health Organization now defines all PPGLs as having metastatic potential, replacing the previous terminology of “malignancy” with “metastatic” (4). Studies show that approximately 10% to 20% of patients with PPGLs develop metastases (2). Survival in metastatic patients has been found to be a median of 6.7 years, and the time between diagnosis and metastatic disease differs greatly, as 1 study found a mean of 43 months but ranging from 0 to 614 months (5). The metastatic potential of PPGL is believed to be correlated with the patient's genotype, and especially correlations between the germline *SDHB* variant and metastatic development have been demonstrated. The association between metastatic disease and other variants remains insufficiently studied (2).

We investigated associations between metastatic development and all genotypes in PPGL patients presented in the literature, as well as the timing of metastases, to better understand the onset of occurrence. This can contribute to optimizing a personalized follow-up program for earlier diagnosis of metastases, thereby increasing treatment options and survival rates.

## Methods

This systematic review is based on the PRISMA (Preferred Reporting Items for Systematic Reviews and Meta-Analyses) workflow. Before commencing the work presented in this paper, a protocol with the registration number CRD42024514279 was published on https://www.crd.york.ac.uk/PROSPERO/.

### Search Strategy and Study Selection

We performed an extensive search in the databases PubMed and Embase on January 29, 2024. The search strategy can be seen in Table S1 (6). Through EndNote 21, the studies were transferred to www.covidence.org for screening, quality assessment, and data extraction. All studies not automatically identified by EndNote 21 were carefully sought through extensive searches conducted within the Royal Danish Library and the respective journal in which the abstract was published. Screening was performed by 2 investigators independently. Conflicts were resolved by collectively reviewing the conflicting abstracts within the investigator team. Full-text retrieval and full-text screening were carried out by the lead investigator.

### Eligibility Criteria

Only studies presenting patients with a PPGL diagnosis and where genetic testing was performed within the cohort were eligible for inclusion. Furthermore, the presence of metastatic disease should be stated according to the genetic status of the patients. It was presumed that the PPGL diagnosis was made based on existing diagnostic guidelines. Data on individuals clinically diagnosed with a familial syndrome such as Von Hippel-Lindau (*VHL*) or neurofibromatosis 1 (*NF1*) were not extracted if confirmatory genetic testing was not conducted. Conforming to the definition by the World Health Organization, metastatic disease was defined as the presence of chromaffin tissue in anatomical sites where it typically does not occur, such as bones, lungs, and liver. This encompasses lymph node spread but not local invasion or recurrence (3). Studies that did not comply with this definition were excluded. Cohorts including only metastatic cases and cohorts with fewer than 10 patients with genetic testing were excluded to minimize reporting and publication bias.

### Quality Assessment

The studies included in the analyses underwent quality assessment. The assessment was based on a modified version of the Newcastle-Ottawa Quality Assessment Scale for cohort studies (7). We assessed low risk of bias if the following criteria were met: (1) selection bias, in the sense that the cohort should be truly representative of the average PPGL patient in the community, meaning an unselected cohort without restrictions to inclusion besides holding a PPGL diagnosis; (2) comparability bias, in that there were no differences in how genetic testing was performed throughout the cohort; and (3) outcome bias, determined by a maximum of 10% lost to follow-up.

### Outcomes and Data Extraction

Our primary outcome was the rate of metastasis in various genetic groups per 100 followed patient-years. We conducted analyses on both individual genetic variants as well as cluster-divided groups. The classification of variants into clusters can be seen in Table S2 (6). Secondary outcomes include the risk ratio of the pooled incidence of metastatic disease in a genetic group compared to the group with no variants identified, as well as the rate of metastatic disease in various genetic groups adjusted for time to metastasis. A data extraction template was manually created in Covidence, and information on germline and somatic mutations in the population, along with their association with metastatic disease, was extracted. When available, time to metastatic disease and follow-up duration were collected. Supplementary data, if provided, were examined to identify relevant information. If multiple studies were published by the same author with similar populations, baseline characteristics and outcomes were compared to identify and exclude duplicates.

### Statistical Analyses

For all outcomes of interest, meta-analyses were performed using a random-effects model with the Paule-Mandel method when at least 3 studies reported the outcome and at least 1 event was observed. Results are presented with 95% confidence intervals (CIs), and statistical heterogeneity was assessed using I^2^. Heterogeneity was considered low when <25%, moderate at 25% to 50%, and high at 50% to 75%. Results were not presented if heterogeneity exceeded 75%. All analyses were conducted in R (version 2023.12.1 + 402).

Rates of metastatic disease per 100 followed patient-years, as well as time to metastasis, were estimated using meta-analyses of single incidence rates. Analyses were performed both for individual genetic variants and for cluster-divided groups. Variant-specific follow-up time was used when available. When only collective follow-up duration for the entire cohort was reported, it was assumed that follow-up did not differ by genetic status, and the overall follow-up time was applied to analyses of individual variants. For time to metastatic disease, only the time explicitly reported for the relevant genetic group was used. Follow-up time and time to metastasis were expressed as means. When time was reported only as medians with interquartile range, the median was converted to a mean using a validated conversion tool incorporating the median, range, and sample size (8). Meta-analyses of time to metastatic disease were conducted both including studies reporting time = 0 and as a sensitivity analysis excluding these studies. For the rate of metastatic disease per 100 followed patient-years, a meta-regression was performed within cluster 1 to assess the effect of *SDHB* on the overall rate.

Meta-analyses of binary outcome data were conducted to calculate the risk ratio of the pooled incidence of metastatic disease in a genetic variant group compared with the group with no identified variant. Studies were eligible for this analysis only if they reported outcomes for both a genetic variant group and a no-identified-variant group.

To explore potential sources of statistical heterogeneity, we conducted subgroup analyses to evaluate the influence of bias (selection, comparability, and outcome) on both the primary outcome (rate of metastatic disease per 100 followed patient-years) and the secondary outcome (risk of metastatic disease in patients with pathogenic germline variants compared to those without) within the *SDHB* group. Furthermore, a meta-regression was performed to examine the impact of publication year on these outcomes. The effect estimates of the moderator variables (publication year and bias) on the respective outcome variables (rate or risk of metastatic disease), along with corresponding *P*-values, were reported. Finally, we generated a funnel plot and a trim-and-fill estimate to explore potential publication bias.

## Results

### Study Selection

**Figure 1.**
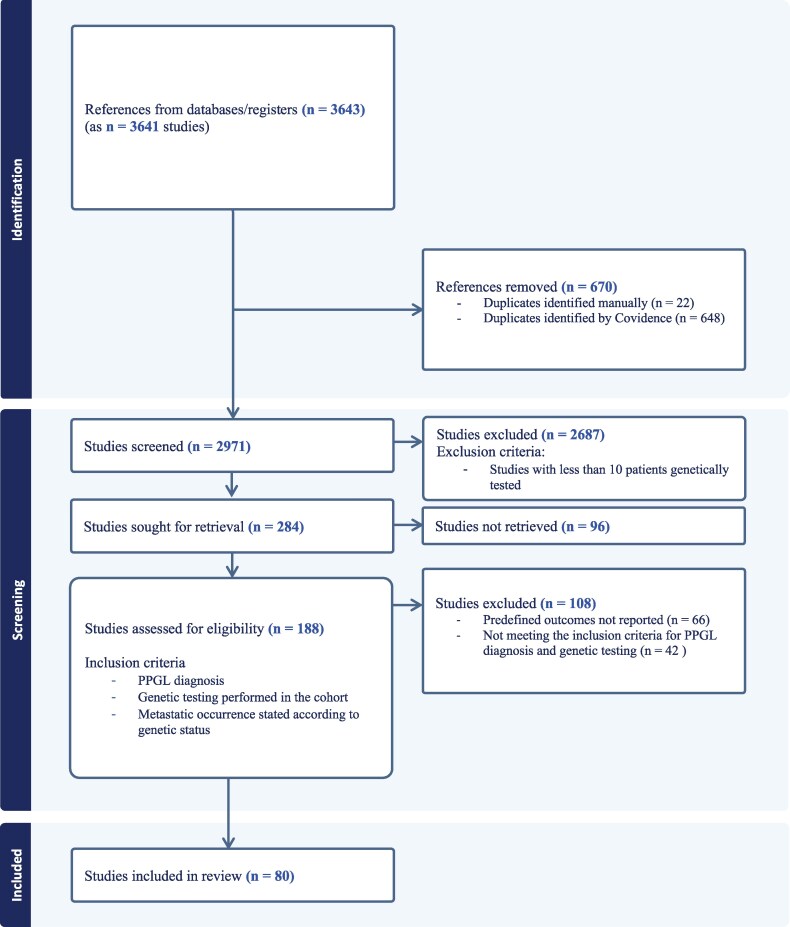
PRISMA flowchart.

Our search identified 3641 studies. Six hundred forty-eight duplicates were automatically removed by the Covidence program and then manually verified. An additional 22 duplicates were identified during the screening process. The remaining 2971 studies were screened by title and abstract by 2 investigators independently. Initial screening left 284 studies for full-text retrieval and screening. Ninety-six studies could not be retrieved. The primary reason for this was the abstracts' origin in symposiums, conferences, or meeting abstracts compilations without corresponding full publication in any accessible source. One hundred eighty-eight studies underwent full-text assessment for eligibility. Sixty-six studies were excluded because the predefined outcome was not reported, and 42 were excluded for not meeting inclusion criteria regarding genetic testing and/or diagnosis, leaving 80 studies for inclusion in the analysis. The selection process is illustrated in the PRISMA flowchart (Fig. 1). The included articles are listed in the reference list (9-88).

### Risk of Bias Assessment

Seventy-four of 80 studies were judged to have a high risk of selection bias, while 6 were considered as truly representative of the average PPGL patient. Thirty-three studies showed a high risk of comparability bias, while 47 genetically tested the entire cohort. Twenty-one studies lost more than 10% of patients to follow-up and were considered at high risk of outcome bias, whereas 59 did not. The risk of bias assessment for the individual studies is presented in Table S3 (6).

### Events of Metastatic Disease in Various Genotypes

**Figure 2.**
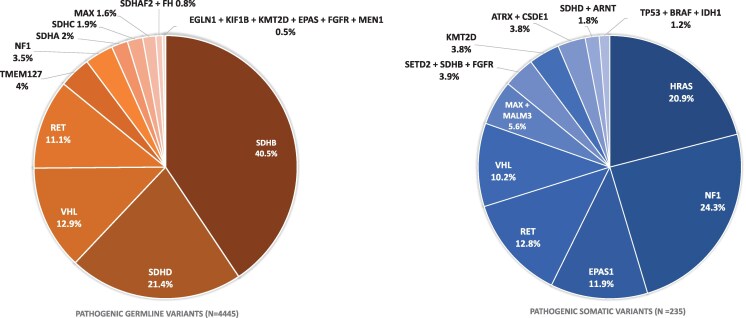
Distribution of pathogenic variants in all paragangliomas and phaeochromocytomas patients. n = total number of pathogenic germline/somatic variants across all included studies.

Through the inclusion of 80 studies, a total of 8972 PPGL patients were included, of whom 1789 (19.9%) had metastatic disease. Incidences of metastatic disease by genetic subgroup are shown in Table 1, while a comprehensive summary of all cohorts is provided in Table S4 (6). In several studies, genetic testing was not performed throughout the entire cohort. A total of 1083 patients with 168 events of metastatic disease (15.5%) were not genetically tested. Genetic testing did not find a pathogenic variant in 3427 patients, of whom 446 experienced metastatic disease (13.0%). The most prevalent variants identified were germline *SDHB* (n = 1799)*, SDHD* (n = 950)*, VHL* (n = 572), and *RET* (n = 492). Twelve studies reported somatic variant analyses, identifying 235 patients with pathogenic somatic variants*. NF1* and *HRAS* were the most frequent, with 57 and 49 cases, respectively. The distribution of pathogenic germline and somatic variants discovered in the included studies is illustrated in Fig. 2. Patients with the germline *SDHB* variant presented as the largest group with metastatic disease, accounting for 926 out of a total of 1789 metastatic events. Notably, they were also the most represented genetic group in general.

**Table 1.** Incidence of metastatic disease by genetic testing results

	Studies n	Number of patients n (% of total) [% of total germline/somatic]	Metastatic disease n (% of total number of patients for the group)
Total	80	8972 (100)	1789 (19.9)
No. genetic testing performed	19	1083 (12.1)	168 (15.5)
Pathogenic variant not identified	51	3427 (38.2)	446 (13.0)
Pathogenic germline variants	79	4445 (49.5) [100]	1152 (25.9)
*SDHA*	18	87 (1.0) [2.0]	14 (16.1)
*SDHB*	66	1799 (20.1) [40.5]	926 (51.5)
*SDHC*	23	86 (1.0) [1.9]	10 (11.6)
*SDHD*	44	950 (10.6) [21.4]	86 (9.1)
*SDHAF2*	5	16 (0.2) [0.4]	1 (6.3)
*FH*	8	18 (0.2) [0.4]	9 (50)
*VHL*	39	572 (6.4) [12.9]	48 (8.4)
*RET*	38	492 (5.5) [11.1]	23 (4.7)
*NF1*	25	157 (1.7) [3.5]	10 (6.4)
*TMEM127*	18	176 (2.0) [4.0]	7 (4.0)
*MAX*	18	73 (0.8) [1.6]	13 (17.8)
*EGLN1*	4	4 (0.04) [0.09]	1 (25)
*KIF1B*	5	7 (0.08) [0.2]	2 (28.6)
*KMT2D*	1	2 (0.02) [0.04]	0 (0)
*EPAS1*	1	2 (0.02) [0.04]	0 (0)
*FGFR*	1	1 (0.01) [0.02]	1 (100)
*MEN1*	3	3 (0.03) [0.1]	1 (33.3)
Pathogenic somatic variants	12	235 (2.6) [100]	24 (10.2)
*HRAS*	7	49 (0.5) [20.9]	3 (6.1)
*RET*	6	30 (0.3) [12.8]	2 (6.7)
*SDHB*	2	3 (0.03) [1.3]	2 (66.7)
*SDHD*	2	2 (0.02) [0.9]	1 (50)
*EPAS1*	9	28 (0.3) [11.9]	4 (14.3)
*NF1*	6	57 (0.6) [24.3]	4 (7.0)
*VHL*	5	24 (0.3) [10.2]	1 (4.2)
*MAX*	3	6 (0.1) [2.6]	0 (0)
*TP53*	1	1 (0.01) [0.4]	0 (0)
*FGFR*	2	3 (0.03) [1.3]	0 (0)
*BRAF*	1	1 (0.01) [0.4]	0 (0)
*ATRX*	1	5 (0.1) [2.1]	3 (60)
*CSDE1*	1	4 (0.04) [1.7]	0 (0)
*SETD2*	1	3 (0.03) [1.3]	2 (66.7)
*ARNT*	1	2 (0.02) [0.9]	0 (0)
*IDH1*	1	1 (0.01) [0.4]	0 (0)
*MAML3*	1	7 (0.1) [3.0]	0 (0)
*KMT2D*	1	9 (0.1) [3.8]	2 (22.2)

### Rate of Metastatic Disease per 100 Followed Patient-years

Overall follow-up duration was reported in 41 studies, ranging from 11 to 312 months. Meta-analysis results for rates of metastatic disease per 100 followed patient-years are displayed in Table 2. For germline variants, significant incidence rates were observed across all genetic groups analyzed. In patients without identified variants, the rate was 1.55. *SDHA, SDHC, SDHD, VHL, RET, NF1*, and *MAX* displayed rates of 13.73, 6.27, 2.03, 2.34, 1.91, 4.11, and 9.66, respectively. The rate for *SDHB* was not presented because statistical heterogeneity exceeded 75%. Pathogenic somatic variants *HRAS, EPAS1, NF1, RET,* and *VHL* were eligible for meta-analysis. *HRAS, RET,* and *VHL* were not presented as statistical heterogeneity exceeded 75%. We performed a sensitivity analysis on *HRAS,* excluding studies reporting time = 0. This is presented in the table as *HRAS* ex. 0. *EPAS1* was the only somatic variant with a significant result, with a rate of 3.82 per 100 followed patient-years.

**Table 2.** Rate of metastatic disease per 100 followed patient-years

Pathogenic variant	Studies (n)	Mean follow-up time (months)	Metastases/total patients (n/n)	Range (lowest to highest metastases/total patients %)	Rate of metastasis per 100 patient-years [95% CI] I^2^
Not identified	21	98.5	78/965	0-42.9	1.55 [1.17; 2.05] I^2^ = 42%
Pathogenic germline variants					
* SDHA*	7	65.6	2/13	0-100	13.73 [5.45; 34.58] I^2^ = 0%
* SDHC*	10	74.6	4/35	0-100	6.27 [2.82; 13.97] I^2^ = 24%
* SDHD*	23	92	39/623	0-66.7	2.03 [1.24; 3.32] I^2^ = 62%
* VHL*	19	96.5	20/229	0-37.5	2.34 [1.46; 3.76] I^2^ = 24%
* RET*	19	100.9	9/240	0-66.7	1.91 [1.05; 3.49] I^2^ = 39%
* NF1*	13	79.7	5/60	0-100	4.11 [2.21; 7.64] I^2^ = 0%
* MAX*	5	69.7	2/8	0-100	9.66 [3.39; 27.55] I^2^ = 0%
* TMEM127*	8	108.1	0/21	0	Not done
* EGLN1*	3	116.6	0/3	0	Not done
Pathogenic somatic variants					
* EPAS1*	5	87.2	1/19	0-16.7	3.82 [1.23; 11.84] I^2^ = 0%
* NF1*	4	114.7	3/51	0-20	0.87 [0.30; 2.47] I^2^ = 0%
* HRAS ex. 0*	4	114.7	1/29	0-5.9	1.67 [0.48; 5.75] I^2^ = 0%
Clusters					
Cluster 1 incl. *SDHB*	109	89.5	506/1854	0-100	4.41 [3.51; 5.55] I^2^ = 73.8%
Cluster 1 excl. *SDHB*	73	89.2	68/948	0-100	3.00 [2.21; 4.09] I^2^ = 46.7%
Cluster 2	54	105	18/364	0-100	­3.1 [2.13; 4.39] I^2^ = 6.7%

Abbreviation: CI, confidence interval.

Cluster-divided analyses showed rates of 4.41 for cluster 1 and 3.1 for cluster 2. Excluding *SDHB* from cluster 1 yielded a rate of 3.0. Meta-regression indicated that the rate was 2.3-fold higher for the *SDHB* variant compared with other cluster 1 variants (rate ratio = 2.29, 95% CI: 1.51; 3.49, *P* = .0001). Meta-analysis for cluster 3 was not possible due to insufficient data.

### Risk Ratios of Metastatic Disease in Variant Groups Compared to no Identified Variant

Meta-analyses comparing the risk of metastatic disease for patients with a pathogenic variant to those without are presented in Table 3 for germline variants and Table 4 for somatic variants. A significantly increased risk of metastasis was found in patients harboring a pathogenic germline variant in *SDHA, SDHB, SDHC, MAX, FH, KIF1B,* and *EGLN1*. The risks were found to be 3.37, 2.66, 2.24, 3.07, 4.5, 6.09, and 2.13, respectively, with corresponding heterogeneity at 0%, 71%, 31%, 65%, 65%, 0%, and 0%. Furthermore, significance was reached for the somatic *EPAS1* variant, displaying a rate of 1.95 with a heterogeneity of 0%.

**Table 3.** Risk of metastatic disease in patients with pathogenic germline variants compared to no pathogenic variant identified

Pathogenic germline variant	Studies n	Metastatic disease/total patients with variant n/n (metastatic disease/total patients with no variant identified n/n)	RR [95% CI] I^2^
*SDHA*	16	9/44 (184/1419)	3.37 [2.33; 4.88] I^2^ = 0%
*SDHB*	46	312/684 (361/2709)	2.66 [1.98; 3.57] I^2^ = 71%
*SDHC*	18	8/57 (193/1577)	2.24 [1.19; 4.22] I^2^ = 31%
*SDHD*	32	33/343 (295/2455)	1.40 [0.87; 2.26] I^2^ = 49%
*SDHAF2*	4	1/15 (59/571)	3.96 [0.70; 22.37] I^2^ = 68%
*RET*	33	16/365 (298/2268)	0.79 [0.48; 1.30] I^2^ = 33%
*VHL*	32	38/361 (300/2218)	1.02 [0.66; 1.57] I^2^ = 37%
*TMEM127*	14	0/22 (205/1620)	Not done
*NF1*	22	9/132 (199/1591)	1.39 [0.78; 2.62] I^2^ = 36%
*MAX*	15	10/37 (227/1475)	3.07 [1.74; 5.42] I^2^ = 65%
*FH*	7	8/17 (207/1337)	4.50 [2.68; 7.53] I^2^ = 65%
*KIF1B*	5	2/7 (58/408)	6.09 [4.47; 8.31] I^2^ = 0%
*EGLN1*	4	1/4 (18/219)	2.13 [1.30; 3.51] I^2^ = 0%
*MEN1*	3	1/3 (59/233)	3.50 [0.61; 20.00] I^2^ = 60%

Abbreviations: CI, confidence interval; RR, risk ratio.

**Table 4.** Risk of metastatic disease in patients with pathogenic somatic variants compared to no pathogenic variant identified

Pathogenic somatic variant	Studies	Metastatic disease/total patients with variant n/n (metastatic disease/total patients with no variant identified n/n)	RR [95% CI] I^2^
*HRAS*	7	3/49 (68/704)	1.39 [0.59; 3.23] I^2^ = 0%
*EPAS1*	9	4/28 (68/659)	1.95 [1.23; 3.10] I^2^ = 0%
*RET*	6	2/30 (53/616)	1.25 [0.49; 3.21] I^2^ = 0%
*VHL*	5	1/24 (27/568)	2.30 [0.77; 6.87] I^2^ = 0%
*NF1*	6	4/57 (18/302)	1.22 [0.47; 3.13] I^2^ = 0%

Abbreviations: CI, confidence interval; RR, risk ratio.

### Time to Metastatic Disease

Time to metastatic disease by genetic variant was reported in 23 studies, ranging from 0 to 240 months, resulting in 5 groups eligible for analysis. Analyses were performed for patients with no pathogenic variant and for germline variants *SDHB, SDHD, VHL,* and *RET*, with 9, 20, 7, 3, and 3 studies included, respectively. Only *VHL* and *RET* analyses showed heterogeneity below 75%, and neither reached significance. Sensitivity analyses excluding studies with a mean time = 0 months (ie, metastatic disease at diagnosis) were performed for *SDHB* and *SDHD*. Heterogeneity remained above 75%. Due to insufficient reported data, no pathogenic somatic variants were eligible for meta-analysis.

### Exploring Statistical Heterogeneity

The primary outcome, rate of metastatic disease per 100 followed patient-years, did not include the *SDHB* variant as heterogeneity exceeded 75%. Meta-regression showed that the rate increased by 0.0917% per publication year (*P* = <.0001). Similarly, the risk ratio of metastatic disease for *SDHB* compared with no genetic variant identified increased by 0.0544% per publication year (*P* = <.0001).

The influence of selection bias on the rate of metastatic disease per 100 followed patient-years within the *SDHB* group could not be assessed, as only 1 study did not show selection bias. Moderator analysis for outcome bias showed no significant difference between bias and no bias [QM(df = 1) = 0.07, *P* = .79]. Analysis for comparability bias showed a significant difference between biased and nonbiased studies [QM(1) = 5.48, *P* = .02]. In biased studies, the effect estimate was 1.69 (95% CI: 1.35-2.02, *P* = <.0001), indicating a significant association between the absence of bias and the rate of metastatic disease. In nonbiased studies, the effect estimate was 2.32 (95% CI: 1.91-2.72, *P* = <.0001), suggesting a stronger association in the presence of bias. No significant differences were found between the various types of bias regarding the risk ratio of metastatic disease in the *SDHB* group compared to the group with no identified genetic variant.

A funnel plot of the *SDHB* group showed some asymmetry in the distributions of studies, suggesting that smaller studies with lower rates of metastatic disease might be underreported or unpublished, indicating potential publication bias. The funnel plot and trim-and-fill estimate are presented in Fig. S1 (6).

## Discussion

Through a systematic review, we analyzed data from 80 studies describing relations between genotype and metastatic disease in PPGL patients. Our primary outcome was the rate of metastatic disease per 100 followed patient-years. All pathogenic germline variants eligible for meta-analysis (*SDHA, SDHC, SDHD, VHL, RET, NF1*, and *MAX*) were associated with a significantly higher rate of metastases compared to patients with no identified variants. *SDHB* was not displayed due to statistical heterogeneity exceeding 75%. Only the somatic variant *EPAS1* demonstrated a significantly increased rate; however, it is essential to note the limited number of patients in these analyses due to the scarcity of studies examining for somatic variants. Cluster-divided analyses revealed metastatic rates of 4.41 and 3.31 per 100 followed patient-years for cluster 1 and cluster 2, respectively. When excluding *SDHB* from cluster 1, the rate fell to 3.0. Notably, the rates for cluster 1 without *SDHB* and cluster 2 were nearly identical at 3.0 vs 3.1. Meta-regression confirmed a statistically significant difference, showing a 2.3-fold higher metastatic rate in *SDHB* patients compared to patients with other cluster 1 variants. These results suggest that the *SDHB* variant is associated with an increased risk of metastases compared to other variants.

We aimed to investigate the time-adjusted risk in greater depth by analyzing time to metastatic disease. However, very few studies provided this information, and the analyses did not yield meaningful results due to very high statistical heterogeneity. In our analyses of risk rate not adjusted for time, we found a significantly increased risk of metastatic disease in patients with pathogenic germline variants in *SDHA, SDHB, SDHC, TMEM127*, *MAX,* and *FH* compared to those with no identified pathogenic variant.

We investigated potential sources of statistical heterogeneity within the *SDHB* group, as this demonstrated the highest degree of heterogeneity in our results while also being the only variant consistently associated with increased metastatic risk in the existing literature. We identified a small but statistically significant effect of publication year on both investigated outcomes. We examined the effect of bias identifying a significant influence of comparability bias on our primary outcome. A funnel plot suggested potential publication bias. While these findings may account for some of the heterogeneity in our results, other contributing factors are likely present.

To the best of our knowledge, this is the most extensive and comprehensive systematic review to date on genetic profiling of PPGLs and associations with metastatic disease. We included studies with very few restrictions, which strengthened scope but introduced some weaknesses. A high degree of heterogeneity in several analyses was found, especially in *SDHB* analyses. Selection bias was prevalent, and also comparability and outcome bias were identified. Analyses of time to metastasis were limited by pervasive bias in the literature in terms of lack of reporting. Also, follow-up time was often not reported. We did not account for treatment interventions or testing modality, which may influence outcomes. Furthermore, we used an overall follow-up duration in specific genetic groups when individual follow-up was not available, as we assumed that there would not be a reason for differentiating follow-up. The increased risk of a bad outcome in *SDHB* carriers is widely recognized and could be a potential reason for a differently structured follow-up program for these patients. Also, most of the published cohorts and, consequently, the included studies originate from highly specialized centers, which tend to receive more complex cases, and this may lead to an overestimation of metastatic risk compared to unselected populations. Moreover, genetic sequencing remains an evolving field, introducing biases through different genetic testing methods and the variants they target. Numerous studies include data spanning several decades, during periods when sequencing technologies were less advanced than they are today. Finally, conclusions regarding the pathogenicity of genetic variants are continuously subject to potential change as ongoing research may lead to corrections.

Presumably, we are the first to perform meta-analyses of the individual genetic alterations' risk of metastatic disease as well as the first to adjust for time. Also, we are seemingly the first to systematically review and analyze associations between somatic variants and metastatic disease. A previous study conducted meta-analyses of cluster-divided groups and *SDHB* independently and found that carrying the *SDHB* germline variant was associated with a higher risk of metastasis. The authors did not find an increased risk in the cluster-divided groups (89). Meta-analyses have also been conducted in the other *SDH* variants, revealing the metastatic disease prevalence of 23% to 31% for *SDHB*, 23% for *SDHC*, 16% for *SDHA,* and 6% to 8% for *SDHD*. We found an almost identical risk rate of *SDHA, -B,* and -*C* and, also consistent with the previous study, a lower risk for *SDHD* compared to the other *SDH* variants (90). An older meta-analysis from 2012 found the pooled incidence of malignant PGL in mutation carriers with manifest disease to be 23% for *SDHB* carriers and 3% for *SDHD* carriers (91). A systematic review of cluster 2 variants found that the prevalence of metastases in cluster 2-related PPGL was 2.6% (2% in *RET*, 5% in *NF1*, 4.8% in *TMEM127,* and 16.7% in *MAX* variants). The authors did not perform meta-analyses (92). We found the prevalence of *RET, NF1,* and *MAX* to be slightly higher at 4.7%, 6.4%, and 17.8%, respectively. Thus, our findings are mostly aligned with previous results on *SDH* variants. Though high heterogeneity excluded results of the individual *SDHB* group, through cluster-divided analyses we confirmed, as seen in the literature, that this variant shows a particularly high metastatic rate. We contribute with the novel suggestion that harboring any genetic variant results in an increased rate of metastatic disease, as demonstrated in both our primary and secondary outcome.

Our work aimed to contribute to the development of individualized clinical guidelines for the management of PPGL patients. Current guidelines have been established by different societies. The North American Neuroendocrine Tumor Society Consensus Guidelines for Surveillance and Management of Metastatic and/or Unresectable Pheochromocytoma and Paraganglioma from 2021 present an association between germline predisposition of a pathogenic *SDHB* variant and increased likelihood of metastatic disease (93). The European Society of Medical Oncology clinical practice guidelines from 2020 suggest assessing the risk of metastatic disease in an individualized manner but taking into account the presence or absence of a germline mutation of a tumor-susceptibility gene. Likewise, they suggest that “high risk of metastases“ can be seen in known *SDHB* germline mutation carriers. They do, however, state that data on long-term follow-up are limited, and follow-up strategies across studies differ (94). A position statement and consensus of the Working Group on Endocrine Hypertension of the European Society of Hypertension from 2020 also state that studies establishing any impact of follow-up on outcomes are scarce. They mention that there is consensus that follow-up should be performed in all patients during the first 10 years after surgery and also that, depending on presentation, there is considerable scope for personalized approaches to follow-up. They also state that patients with large tumors, paragangliomas, a childhood presentation, multifocal disease, or *SDHB* mutation have a higher risk of metastatic progression and require lifelong follow-up (2). Our findings support the existing notion that *SDHB* is associated with a particularly high risk of metastases and that this risk is presumably greater than for other variants, as demonstrated in our cluster-divided analyses. We observed that harboring any pathogenic genetic variant is associated with a higher rate of developing metastases compared to individuals with no identified variant, highlighting the need for more frequent and meticulous follow-up in these patients. Additional research in the form of large cohort studies with low risk of selection bias, performing extensive genetic testing and presentation of follow-up information, as well as time to metastatic disease, is, however, needed. Also, the interplay between genetic profile and other factors such as catecholamine profile, tumor-specific features, etc. in the development of metastatic disease needs to be examined.

## Conclusion

We present a systematic review and meta-analyses on the risk of metastatic disease for all pathogenic genetic variants in PPGL patients reported in the current literature. We examined the timing of metastases by performing meta-analyses on the rate of metastatic disease per 100 followed patient-years. Germline *SDHA, SDHC, SDHD, VHL, RET, NF1*, and *MAX,* as well as somatic *EPAS1,* all exhibited higher rates compared to patients who did not harbor a pathogenic genetic variant. Individual rate for germline *SDHB* was not presented due to very high statistical heterogeneity. In cluster-divided analyses, both cluster 1 and cluster 2 demonstrated higher rates of metastatic disease compared to individuals without identified variants. Meta-regression demonstrated that germline *SDHB* significantly increased this rate relative to the other pathogenic genetic variants in cluster 1. Overall, our findings indicate that carrying any pathogenic genetic variant is associated with a higher rate of metastases compared to individuals with no identified variant; however, the high degree of heterogeneity should be considered. Additional large-scale cohort studies remain essential.

**Human Genes:**  *ARNT*, aryl hydrocarbon receptor nuclear translocator; *ATRX*, alpha thalassemia/mental retardation syndrome X-linked; *BRAF*, v-Raf murine sarcoma viral oncogene homolog B; *CSDE1*, cold shock domain containing E1; *EGLN1*, egg-laying defective nine (egl-9) family hypoxia-inducible factor 1 gene; *EPAS1*, endothelial PAS domain protein 1; *FGFR*, fibroblast growth factor receptor; *FH*, fumarate hydratase; *HRAS*, Harvey rat sarcoma viral oncogene homolog; *IDH1*, isocitrate dehydrogenase 1; *KIF1B*, kinesin family member 1B; *KMT2D*, lysine (K)-specific methyltransferase 2D; *MAML3*, mastermind like transcriptional coactivator 3; *MAX*, MYC-associated factor X; *MEN1*, multiple endocrine neoplasia type 1; *NF1*, neurofibromatosis type 1; *RET*, rearranged during transfection; *SDH*, succinate dehydrogenase subunit A, B, C, and D; *SDHAF2*, succinate dehydrogenase complex assembly factor-2; *SET2D*, SET domain containing 2; *TMEM127*, transmembrane domain protein 127; *TP53*, tumor protein P53; *VHL*, Von Hippel-Lindau.

## Data Availability

All original data generated and analyzed in this study are included in this article or in the supplementary material.
